# High expression of transcriptional coactivator p300 correlates with aggressive features and poor prognosis of hepatocellular carcinoma

**DOI:** 10.1186/1479-5876-9-5

**Published:** 2011-01-05

**Authors:** Mei Li, Rong-Zhen Luo, Jie-Wei Chen, Yun Cao, Jia-Bin Lu, Jie-Hua He, Qiu-Liang Wu, Mu-Yan Cai

**Affiliations:** 1 State Key Laboratory of Oncology in South China, Sun Yat-Sen University Cancer Center, Guangzhou, PR China; 2 Department of Pathology, Sun Yat-Sen University Cancer Center, Guangzhou, PR China

## Abstract

**Background:**

It has been suggested that p300 participates in the regulation of a wide range of cell biological processes and mutation of p300 has been identified in certain types of human cancers. However, the expression dynamics of p300 in hepatocellular carcinoma (HCC) and its clinical/prognostic significance are unclear.

**Methods:**

In this study, the methods of reverse transcription-polymerase chain reaction (RT-PCR), Western blotting and immunohistochemistry (IHC) were utilized to investigate protein/mRNA expression of p300 in HCCs. Receiver operating characteristic (ROC) curve analysis, spearman's rank correlation, Kaplan-Meier plots and Cox proportional hazards regression model were used to analyze the data.

**Results:**

Up-regulated expression of p300 mRNA and protein was observed in the majority of HCCs by RT-PCR and Western blotting, when compared with their adjacent non-malignant liver tissues. According to the ROC curves, the cutoff score for p300 high expression was defined when more than 60% of the tumor cells were positively stained. High expression of p300 was examined in 60/123 (48.8%) of HCCs and in 8/123 (6.5%) of adjacent non-malignant liver tissues. High expression of p300 was correlated with higher AFP level, larger tumor size, multiplicity, poorer differentiation and later stage (*P *< 0.05). In univariate survival analysis, a significant association between overexpression of p300 and shortened patients' survival was found (*P *= 0.001). In different subsets of HCC patients, p300 expression was also a prognostic indicator in patients with stage II (*P *= 0.007) and stage III (*P *= 0.011). Importantly, p300 expression was evaluated as an independent prognostic factor in multivariate analysis (*P *= 0.021). Consequently, a new clinicopathologic prognostic model with three poor prognostic factors (p300 expression, AFP level and vascular invasion) was constructed. The model could significantly stratify risk (low, intermediate and high) for overall survival (*P *< 0.0001).

**Conclusions:**

Our findings provide a basis for the concept that high expression of p300 in HCC may be important in the acquisition of an aggressive phenotype, suggesting that p300 overexpression, as examined by IHC, is an independent biomarker for poor prognosis of patients with HCC. The combined clinicopathologic prognostic model may become a useful tool for identifying HCC patients with different clinical outcomes.

## Background

Hepatocellular carcinoma (HCC) is the fifth most common cancer in the world and the third leading cause of cancer mortality [1]. It is among the top three causes of cancer death in the Asian Pacific region due to the high prevalence of chronic hepatitis B virus and hepatitis C virus infections, and recently its incidence in the United States and in Western Europe has been increasing [2,3]. Despite new therapies and attempts for early detection of primary HCC, the long-term survival of HCC patient remains poor. Surgery is considered as one of the standard curative treatments for HCC if the tumor is resectable [4]. However, the prognosis of HCC patients with the same clinical stage often differs substantially in spite of curative surgical resection and such large variation is mostly unexplained. Thus, a large amount of investigations on HCC have focused on the discovery of specific molecular markers that could serve as reliable prognostic factors. To date, however, the search for specific molecules in HCC cells that have clinical/prognostic value remains substantially limited.

Recently, it has been reported that p300, a member of the histone acetyltransferase family of transcriptional coactivator, is found to play a variety of roles in the transcription process and catalyzes histone acetylation through its histone acetyltransferase activity [5,6]. Transcriptional coactivator p300 has been shown to participate in the regulation of various cellular processes such as proliferation, differentiation, apoptosis, cell-cycle regulation and DNA damage response [7]. A tumor suppressor role of p300 has been identified in certain types of human cancers, including breast, colorectal and gastric carcinoma [8,9]. However, several studies suggest that transcriptional coactivator p300 is a positive regulator of cancer progression and related to tumorigenesis of various human cancers [10,11]. The translational co-activator p300 was found to be involved in the integrin beta-1-mediated histone acetylation and p21 transcriptional activation in HCC [12]. In addition, Wang et al [13] suggested that a direct role of phosphor-CREB in p300 and Brg I recruitment to the *Hulc *promoter led to the activation of epigenetic markers and chromatin remodeling at the same location in hepatic cancer cells. It has also been reported that p300 expression correlates with nuclear alterations of tumor cells and contributes to the growth of prostate carcinoma and is a predictor of aggressive features of this cancer [14,15].

Up to date, the clinicopathologic/prognostic implication of p300 in HCC has not been explored. In this study, reverse transcription-polymerase chain reaction (RT-PCR), Western blotting, immunohistochemistry (IHC) and tissue microarray were utilized to examine the distribution and frequency of p300 expression in our HCC cohort and adjacent non-malignant liver tissues. In order to avoid predetermined cutpoint, receiver operating characteristic (ROC) curve analysis was employed to define the cutoff score for high expression of p300. In addition, the correlation between p300 expression and cell proliferation levels in our HCCs was analyzed using the Ki-67 assessment marker.

## Methods

### Patients and tissue specimens

In this study, the paraffin-embedded pathologic specimens from 123 patients with HCC were obtained from the archives of Department of Pathology, Sun Yat-Sen University Cancer Center, Guangzhou, China, between July 2005 and May 2008. The cases selected were based on distinctive pathologic diagnosis of HCC, undergoing primary and curative resection for tumor without preoperative anticancer treatment, availability of resection tissue and follow-up data. These HCC cases included 107 (87.0%) men and 16 (13.0%) women, with mean age of 47.7 years. Average follow-up time was 26.79 months (median, 28.0 months; range, 1.0 to 61 months).

Patients whose cause of death remained unknown were excluded from our study. Clinicopathologic characteristics for these patients including age, sex, hepatitis history, alpha-fetoprotein (AFP), liver cirrhosis, tumor number, size, differentiation, stage, vascular invasion and relapse were detailed in Table 1. Tumor differentiation was based on the criteria proposed by Edmonson and Steiner [16]. Tumor stage was defined according to American Joint Committee on Cancer/International Union Against Cancer tumor-node-metastasis (TNM) classification system [17]. Institute Research Medical Ethics Committee of Sun Yat-Sen University Cancer Center granted approval for this study.

**Table 1:** Correlation of p300 expression with patients' clinicopathologic features in primary hepatocellular carcinomas

	p300 protein			
	
Variable	All cases	Low expression	High expression	***P *value**^a^
Age (years)				0.267
≤ 47.7^b^	59	28 (47.5%)	31 (52.5%)	
>47.7	64	35 (54.7%)	29 (45.3%)	
Sex				0.564
Male	107	55 (51.4%)	52 (48.6%)	
Female	16	8 (50.0%)	8 (50.0%)	
Etiology				0.295
HBV	97	48 (49.5%)	49 (50.5%)	
HCV	8	3 (37.5%)	5 (62.5%)	
None	18	12 (66.7%)	6 (33.3%)	
AFP (ng/ml)				0.000
≤ 20	68	46 (67.6%)	22 (32.4%)	
>20	55	17 (30.9%)	38 (69.1%)	
Liver cirrhosis				0.334
Yes	87	47 (54.0%)	40 (46.0%)	
No	36	16 (44.4%)	20 (55.6%)	
Tumor size (cm)				0.000
≤ 5	76	50 (65.8%)	26 (34.2%)	
>5	47	13 (27.7%)	34 (72.3%)	
Tumor multiplicity				0.012
Single	85	50 (58.8%)	35 (41.2%)	
Multiple	38	13 (34.2%)	25 (65.8%)	
Differentiation				0.036
Well	15	12 (80.0%)	3 (20.0%)	
Moderate	70	36 (51.4%)	34 (48.6%)	
Poor	32	14 (43.8%)	18 (56.3%)	
Undifferentiated	6	1 (16.7%)	5 (83.3%)	
Stage				0.015
I	12	10 (83.3%)	2 (16.7%)	
II	49	27 (55.1%)	22 (44.9%)	
III	48	23 (47.9%)	25 (52.1%)	
IV	14	3 (21.4%)	11 (78.6%)	
Vascular invasion				0.130
Yes	55	24 (43.6%)	31 (56.4%)	
No	68	39 (57.4%)	29 (42.6%)	
Relapse				0.182
Yes	42	18 (42.9%)	24 (57.1%)	
No	81	45 (55.6%)	36 (44.4%)	
Ki67 expression				0.002
Low	68	44 (64.7%)	24 (35.3%)	
High	50	18 (36.0%)	32 (64.0%)	

^a^Chi-square test; ^b^Mean age; HBV, hepatitis B virus; HCV, hepatitis B virus; AFP, alpha-fetoprotein.

### RT-PCR

Total RNA was isolated from 8 pairs of HCC tissues and adjacent non-malignant liver tissues using TRIZOL reagent (Invitrogen, Carlsbad, CA). RNA was reverse-transcribed using SuperScript First Strand cDNA System (Invitrogen, Carlsbad, CA) according to the manufacture's instructions. PCR was performed as described previously using specific primers for p300 [18]. The expression of GAPDH was monitored as a control.

### Western blotting analysis

Equal amounts of whole cell and tissue lysates were resolved by SDS-polyacrylamide gel electrophoresis (PAGE) and electrotransferred on a polyvinylidene difluoride (PVDF) membrane (Pall Corp., Port Washington, NY). The tissues were then incubated with primary mouse monoclonal antibodies against human anti-p300 (Abcam, Cambridge, MA) at a concentration of 0.5 μg/ml. The immunoreactive signals were detected with enhanced chemiluminescence kit (Amersham Biosciences, Uppsala, Sweden). The procedures followed were conducted in accordance with the manufacturer's instructions.

### Tissue microarray (TMA) construction

Tissue microarray was constructed as the method described previously [19]. In brief, formalin-fixed, paraffin-embedded tissue blocks and the corresponding H&E-stained slides were overlaid for TMA sampling. The slides were reviewed by a senior pathologist (M-Y. C.) to determine and mark out representative tumor areas. Triplicates of 0.6 mm diameter cylinders were punched from representative tumor areas and from adjacent non-malignant liver tissue of individual donor tissue block and re-embedded into a recipient paraffin block at defined position, using a tissue arraying instrument (Beecher Instruments, Silver Spring, MD). The TMA block contained 126 HCCs and adjacent non-malignant liver tissues.

### Immunohistochemistry (IHC)

The TMA slides were dried overnight at 37°C,deparaffinized in xylene, rehydrated through graded alcohol, immersed in 3% hydrogen peroxide for 20 minutes to block endogenous peroxidase activity, and antigen-retrieved by pressure cooking for 3 minutes in ethylenediamine tetraacetic acid (EDTA) buffer (pH = 8.0). Then the slides were preincubated with 10% normal goat serum at room temperature for 30 minutes to reduce nonspecific reaction. Subsequently, the slides were incubated with mouse monoclonal anti-p300 (Abcam, Cambridge, MA) at a concentration of 3 ng/ml and mouse monoclonal anti-Ki-67 (Zymed Laboratories Inc., South San Francisco, CA, 1:100 dilution) for 2 hours at room temperature. The slides were sequentially incubated with a secondary antibody (Envision; Dako, Glostrup, Denmark) for 1 hour at room temperature, and stained with DAB (3,3-diaminobenzidine). Finally, the sections were counterstained with Mayer's hematoxylin, dehydrated, and mounted. A negative control was obtained by replacing the primary antibody with a normal murine IgG. Known immunostaining positive slides were used as positive controls.

### IHC evaluation

Nuclear immunoreactivity for p300 protein was reported in semi-quantitative method by evaluating the number of positive tumor cells over the total number of tumor cells. Scores were assigned by using 5% increments (0%, 5%, 10%-100%). Expression for p300 was scored by 3 independent pathologists (M. L., R-Z. L. and M-Y. C.) blinded to clinicopathologic data. Their conclusions were in complete agreement in 82.1% of the cases, which identified this scoring method as highly reproducible.

### Selection of Cutoff Score

**Figure 1:**
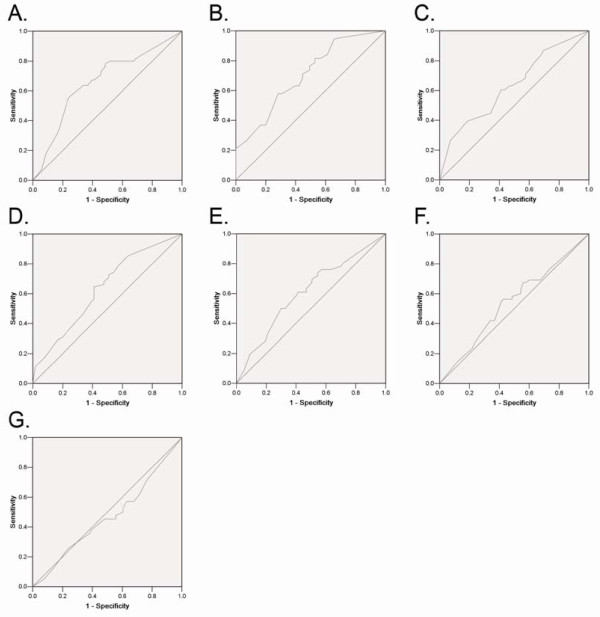
**ROC curve analysis was created to determine the cutoff score for high expression of p300 protein**. The sensitivity and specificity for each outcome were plotted: AFP level (A.), tumor size (B.), tumor multiplicity (C.), tumor differentiation (D.), clinical stage (E.), vascular invasion (F.), tumor relapse(G.).

ROC curve analysis was employed to determine cutoff score for tumor "high expression" by using the 0,1-criterion [20]. At the p300 score, the sensitivity and specificity for each outcome under study was plotted, thus generating various ROC curves (Figure 1). The score was selected as the cutoff value, which was closest to the point with both maximum sensitivity and specificity. Tumors designated as "low expression" for p300 were those with scores below or equal to the cutoff value, while "high expression" tumors were those with scores above the value. In order to use ROC curve analysis, the clinicopathologic features were dichotomized: AFP level (≤ 20 ng/ml or >20 ng/ml), tumor size (≤ 5 cm or >5 cm), tumor multiplicity (single or multiple), tumor grade (well-moderately or poorly-undifferentiated), stage (I + II or III + IV), vascular invasion (absence or presence), relapse (absence or presence) and survival status (death due to HCC or censored).

### Statistical analysis

Statistical analysis was performed by using the SPSS statistical software package (standard version 13.0; SPSS, Chicago, IL). ROC curve analysis was applied to determine the cutoff score for high expression of p300 and Ki67. The correlation between p300 expression and clinicopathologic features of HCC patients was evaluated by χ^2^-test. Univariate and multivariate survival analyses were performed using the Cox proportional hazards regression model. Survival curves were obtained with the Kaplan-Meier method. Predictive accuracy was quantified using the Harrell concordance index. Differences were considered significant if the *P*-value from a two-tailed test was <0.05.

## Results

### *p300 *mRNA expression examined by RT-PCR and p300 protein expression by Western blotting in liver tissues

**Figure 2:**
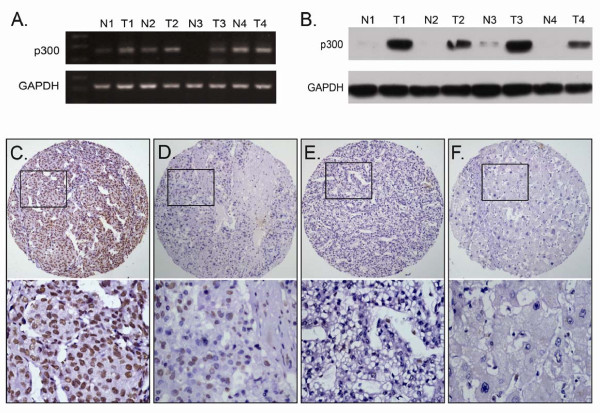
**The mRNA and protein expression of p300 in HCC and adjacent non-malignant liver tissues**. A. Up-regulated expression of *p300 *mRNA was examined by RT-PCR in 3/4 HCC cases, when compared with adjacent non-malignant liver tissues. B. Up-regulated expression of p300 protein was detected by Western blotting in 4/4 HCC cases, when compared with adjacent non-malignant liver tissues. C. High expression of p300 was observed in a HCC (case 26), in which more than 90% tumor cells revealed positive immunostaining of p300 in nuclei (*upper panel*, × 100). D. A HCC case (case 81) demonstrated low expression of p300, in which less than 50% of tumor cells showed immunoreactivity of p300 protein in nuclei (*upper panel*, × 100). E. Nearly negative expression of p300 protein was demonstrated in a HCC case (case 57, *upper panel*, × 100). F. The adjacent non-malignant liver tissues of HCC case 26 showed nearly negative expression of p300 protein (*upper panel*, × 100). The *lower panels *indicated the higher magnification (× 400) from the area of the box in C., D., E. and F., respectively.

In this study, the status of expression of *p300 *mRNA and p300 protein was further examined by RT-PCR and Western blotting, respectively, in 8 pairs of fresh HCC and adjacent non-tumorous liver specimens. The results showed that a total of 5/8 (62.5%) HCCs was examined as having up-regulated *p300 *mRNA expression, when compared with their adjacent non-malignant liver tissues (Figure 2A). Up-regulated expression of p300 protein was observed in 6/8 (75.0%) HCCs, and in each of the four cases with up-regulated p300 protein, up-regulated *p300 *mRNA was observed (Figure 2B).

### The expression of p300 in HCC and adjacent non-malignant liver tissues by IHC

For p300 IHC staining in HCCs and adjacent non-malignant liver tissues, immunoreactivity was primarily observed in the nuclei within tumor cells (Figure 2C). p300 expression could be evaluated informatively in 123 HCCs by the TMA constructed previously. The non-informative 3 TMA samples included samples with too few tumor cells (<300 cells per case) and lost samples. Immunoreactivity of p300 in HCC ranged from 0% to 100% (Figure 2C-2F). According to ROC curve analysis, expression percentage for p300 above the cutoff value 60% was defined as high expression, while below or equal to the cutoff value was considered as low expression. In this study, 16 of the 123 (13.0%) HCC samples showed completely negative staining of p300. High expression of p300 could be detected in 60/123 (48.8%) of HCCs, in 6/87 (6.9%) of adjacent liver tissues with cirrhosis and in 2/36 (5.6%) of adjacent normal liver tissues without cirrhosis, respectively (*P *< 0.0001, Fisher's exact test).

### Selection of cutoff scores for p300 expression

The ROC curves for each clinicopathological parameter (Figure 1) clearly show the point on the curve closest to (0.0, 1.0) which maximizes both sensitivity and specificity for the outcome as described in our previous study [19]. Tumors with scores above the obtained cutoff value were considered as high p300 expression leading to the greatest number of tumors classified based on clinical outcome presence or absence. The corresponding area under the curve (AUC, 95% CI) were collected and listed in Table 2. Cutoff score for p300 high expression was determined to be more than 60% carcinoma cells staining.

**Table 2:** Area under the curve (AUC) of receiver operating characteristic curve for each clinicopathologic feature

Variable	AUC (95% CI)	*P *value
AFP	0.662 (0.563 to 0.760)	0.002
Tumor size	0.703 (0.606 to 0.800)	0.000
Tumor multiplicity	0.633 (0.525 to 0.741)	0.019
Differentiation	0.634 (0.536 to 0.732)	0.010
Stage	0.609 (0.505 to 0.713)	0.044
Vascular invasion	0.544 (0.441 to 0.647)	0.407
Relapse	0.466 (0.357 to 0.576)	0.543

CI indicates confidence interval.

### Association of p300 expression with HCC patients' clinicopathological parameters

**Figure 3:**
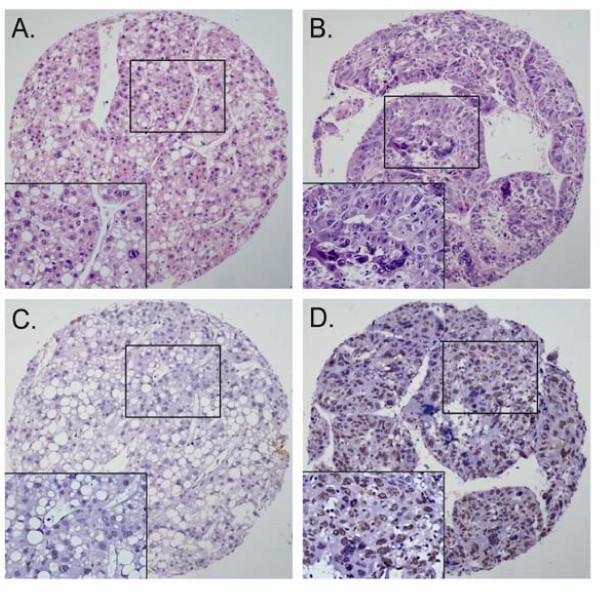
**The altered expression levels of p300 in HCC tissues by immunohistochemistry**. A. and B. represented H&E staining for well-differentiated HCC (case 43) and poorly-differentiated HCC (case 37), respectively. C. Low expression of p300 was observed in a well-differentiated HCC case (case 43), in which less than 5% of tumor cells showed immunoreactivity of p300 protein in nuclei (×100). D. High expression of p300 was demonstrated in the poor-differentiated HCC case (case 37), in which more than 60% carcinoma cells showed immunoreactivity of p300 in nuclei (×100). Representative sites in HCC tissue with higher (inset, ×400) magnification were shown.

The high or low expression rates of p300 in HCCs with respect to several standard clinicopathologic features are presented in Table 1. The high p300 expression rate was higher in patients with higher AFP levels (*P *< 0.0001), larger tumor size (*P *< 0.0001), tumor multiplicity (*P *= 0.012), poorer differentiation (*P *= 0.036, Table 1, Figure 3) and later stage (*P *= 0.015, Table 1). There was no significant correlation between p300 expression and other clinicopathologic parameters, such as patient age (≤47.7 years *vs *>47.7 years), sex, hepatitis history, liver cirrhosis, tumor vascular invasion and relapse (*P *> 0.05, Table 1).

### Relationship between clinicopathologic features, p300 expression, and HCC patients' survival: Univariate survival analysis

**Figure 4:**
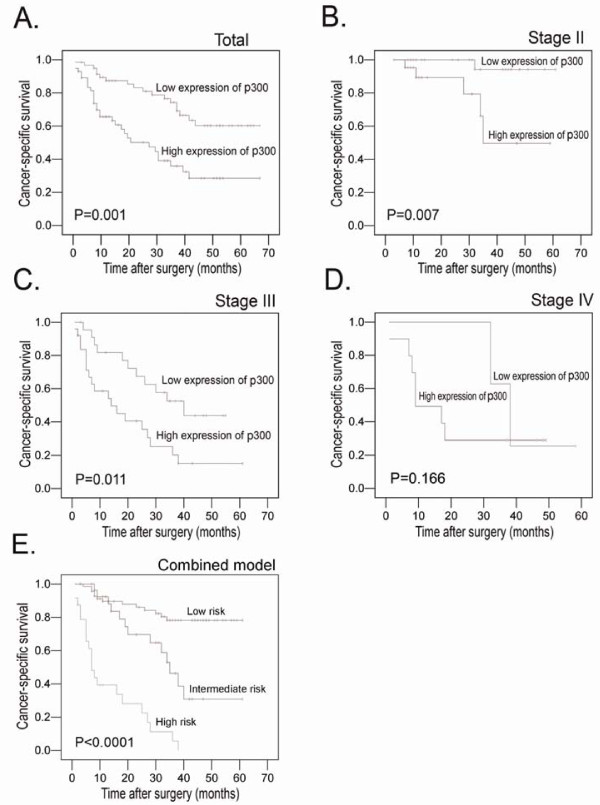
**Kaplan-Meier survival analysis of p300 expression in total patients and subsets of different stage patients with HCC (log-rank test)**. A. *Total*, probability of survival of all patients with HCC: low expression, n = 63; high expression, n = 60. B. *Stage II*, probability of survival of stage II patients with HCC: low expression, n = 27; high expression, n = 22. C. *Stage III*, probability of survival of stage III patients with HCC: low expression, n = 23; high expression, n = 25. D. *Stage IV*, probability of survival of stage IV patients with HCC: low expression, n = 3; high expression, n = 11. E. Comparison of overall survival according to a new combined clinicopathologic prognostic model (including p300, AFP level and vascular invasion): low risk, n = 70; intermediate risk, n = 29; high risk, n = 24.

In order to confirm the representativeness of the HCCs in our study, we analyzed established prognostic factors of patients' survival. Kaplan-Meier analysis demonstrated a significant impact of well-known clinicopathologic prognostic parameters, such as serum AFP levels (*P *< 0.0001), tumor size (*P *< 0.0001), tumor multiplicity (*P *< 0.0001), clinical stage (*P *< 0.0001), vascular invasion (*P *< 0.0001), and relapse (*P *< 0.0001) on patients' survival (Table 3). Assessment of survival in total HCCs revealed that high expression of p300 was correlated with adverse disease-specific survival of HCC patients (*P *= 0.001, Table 3, Figure 4A). Further analysis was performed with regard to p300 expression in subsets of patients with different stages. The results demonstrated as well that high expression of p300 was a prognostic factor in HCC patients with stage II (*P *= 0.007, Figure 4B) and stage III (*P = *0.011, Figure 4C). However, it could not differentiate the outcome of stage I (not reached) or stage IV patients (*P *= 0.166, Figure 4D).

**Table 3:** Univariate and multivariate analysis of different prognostic factors in 123 patients with hepatocellular carcinoma (Cox Proportional Hazards Regression)

	Univariate analysis			Multivariate analysis	
**Variable**	**All cases**	**HR (95% CI)**	***P *value**	**HR (95% CI)**	***P *value**

Age (years)			0.883		
≤47.9^a^	59	1.0			
>47.9	64	1.044 (0.588-1.853)			
Sex			0.746		
Male	107	1.153 (0.489-2.717)			
Female	16	1.0			
Hepatitis history			0.806		
Yes	105	0.904 (0.405-2.021)			
No	18	1.0			
AFP (ng/ml)			0.000		0.014
≤20	68	1.0		1.0	
>20	55	5.445 (2.852-10.395)		2.573 (1.209-5.476)	
Liver cirrhosis			0.807		
Yes	87	1.0			
No	36	1.082 (0.578-2.026)			
Tumor size (cm)			0.000		0.167
≤5	76	1.0		1.0	
>5	47	2.946 (1.640-5.290)		1.595 (0.823-3.090)	
Tumor multiplicity			0.000		0.077
Single	85	1.0		1.0	
Multiple	38	3.768 (2.108-6.735)		1.790 (0.939-3.414)	
Differentiation			0. 099		
Well-moderate	85	1.0			
Poor-undifferentiated	38	1.642 (0.911-2.958)			
Stage			0.000		0.363
I-II	61	1.0		1.0	
III -IV	62	5.828 (2.722-12.480)		1.571 (0.593-4.162)	
Vascular invasion			0.000		0.015
Yes	55	5.372 (2.724-10.595)		2.724 (1.214-6.113)	
No	68	1.0		1.0	
Relapse			0.000		0.321
Yes	42	2.885 (1.608-5.174)		1.390 (0.725-2.666)	
No	81	1.0		1.0	
p300			0.001		0.021
Low expression	63	1.0		1.0	
High expression	60	2.792 (1.533-5.087)		2.077 (1.149-4.112)	
Ki67			0.089		
Low expression	68	1.0			
High expression	50	1.661 (0.925-2.982)			

^a^Mean age; AFP, alpha-fetoprotein; HR, hazards ratio; CI, confidence interval.

### Independent prognostic factors of HCC: Multivariate Cox regression analysis

Since features observed to have a prognostic influence by univariate analysis may covariate, p300 expression and those clinicopathologic variables that were significant in univariate analysis (i.e., AFP levels, tumor size, tumor multiplicity, clinical stage, vascular invasion, and relapse) were further examined in multivariate analysis. Results showed that high expression of p300 was an independent prognostic factor for poor patient overall survival (hazard ratio, 2.077; 95%CI, 1.149-4.112, *P *= 0.021; Table 3). Of the other parameters, serum AFP level (*P *= 0.014) and vascular invasion (*P *= 0.015) were evaluated as well independent prognostic factors for patients' overall survival.

### Prognostic model with p300 expression, AFP level and vascular invasion

According to the results of our univariate and multivariate analyses, we proposed a new clinicopathologic prognostic model with three poor prognostic factors: p300 expression, AFP level and vascular invasion. Thus, we designated a high-risk group as the presence of the three factors (including p300 expression, AFP level and vascular invasion), an intermediate-risk group as the presence of two factor (regardless of their identity), and a low-risk group as the presence of one factor or none. The model could significantly stratify risk (low, intermediate and high) for overall survival based upon a combination of p300 and the standard clinicopathologic features (*P *< 0.0001, Figure 4E). In addition, application of Harrell concordance index to the proposed new clinicopathologic prognostic model showed improved predictive ability when compared with the standard pathological feature model (c indexes of 0.689 vs 0.648, respectively).

### Correlation between p300 expression and cell proliferation in HCCs

To address whether or not p300 expression in HCC is correlated with cell proliferation, the expression of Ki-67, a widely used cellular proliferation marker, was investigated by IHC in our HCC cohort. Among the 123 HCCs, in 118 samples, p300 and Ki-67 IHC were examined successfully and simultaneously. According to the ROC curve analysis, the cutoff score for Ki67 high expression was determined to be more than 50% carcinoma cells staining (data not shown). Using this designation, high expression of Ki67 was detected in 50/118 (42.4%) HCCs. In addition, a significant positive correlation between expression of p300 and Ki67 was evaluated in our HCC cohort, in which the frequency of cases with high expression of Ki67 was significantly larger in carcinomas with a high expression of p300 (32/56 cases, 57.1%) than in those cases with a low expression of p300 (18/62 cases, 29.0%) (*P *= 0.002, Table 1).

## Discussion

Transcriptional coactivator p300 has the potential to participate in a variety of cellular functions, such as cell proliferation and differentiation, senescence and apoptosis [7]. Recently several studies have documented an involvement of p300 in oncogenic processes, such as lung, colon, prostate, breast cancer and leukemia [14,21-24]. However, the status of p300 and its potential prognostic impact on HCC have not been explored so far. In the present study, we examined the expression levels of *p300 *mRNA and p300 protein in HCC tissues and adjacent non-malignant liver tissues, firstly by RT-PCR and Western blotting. Our results established that up-regulated expression of *p300 *mRNA and p300 protein was shown in the majority of HCCs, when compared with their adjacent non-malignant liver tissues. Subsequently, the expression dynamics of p300 protein was investigated by IHC, using a TMA containing HCC tissues and adjacent non-malignant liver tissues. Our IHC results demonstrated that high expression of p300 was more frequently observed in HCC tissues when compared to the adjacent liver tissues with or without cirrhosis. The expression of p300 in adjacent non-malignant liver tissues with or without cirrhosis was either absent or at low levels. In contrast, in large number of our HCC tissues, high expression of p300 was frequently observed. Previous studies also described that mutation in *p300 *gene, accompanied by loss of the other allele, was observed in certain types of tumors, including colorectal, gastric and breast cancers [8,9]. In addition, the frequency of promoter methylation of *p300 *gene was found in 65.8% of HCC [25]. These findings provide evidence that the up-regulation of p300 may play an important role in tumorigenic process of HCC.

To assess the significance of p300 protein in HCC and avoid predetermined arbitrary cutpoint, ROC curve analysis was applied to determine cutoff score for p300 expression as described in our previous study [19]. Further correlation analysis revealed that high expression of p300 in HCCs was correlated with higher serum AFP levels, larger tumor size, tumor multiplicity, poorer differentiation and later clinical stage. Importantly, high expression of p300 was a strong and independent predictor of shortened overall survival as evidenced by univariate and multivariate analysis. In addition, stratified survival analysis of HCC accordingly to clinical stage evaluated p300 expression to be closely correlated with survival of HCC patients with stage II or stage III. Since a relatively less cases of HCC were included in stage I or stage IV, we did not found statistically significant correlation for these HCC-subgroups in univariate analysis. Our findings in this study suggest that expression of p300 in HCC may facilitate an increased malignant feature and/or worse prognosis of this tumor. Previous study also suggested that putative p300 and CREB complex might up-regulate the H3 and H4 acetylation levels, and then up-regulated the Hulc expression level which was identified as the most important genes in HCC [13]. Thus, the examination of p300 expression by IHC could be used as an additional tool in identifying those patients at risk of HCC progression; p300 expression analysis may also be useful in optimizing individual HCC therapy management: favoring a more aggressive regimen in tumors with a high expression of p300.

Although several characteristics of CBP and p300 suggested that these proteins might serve as tumor suppressors, some studies reported an important role of p300 protein in oncogenic processes [7,26]. In prostate cancer, p300 expression was shown to be linked to proliferation and identified as a predictor of progression of this cancer [14]. In colon carcinoma, overexpression of p300 was an indicator of poor prognosis [21]. Moreover, p300 mRNA levels were observed to correlate with lymph node status in breast cancer [24]. However, p300 protein levels did not show significant correlations with tumor grade or nodal positivity in other study [27,28]. In the present study, we did observe that high expression of p300 was associated with an aggressive feature of HCC and was a strong and independent predictor of shorter cancer-specific survival. Considering that the mechanism by which coactivator p300 promotes gene transcription may vary among gene targets, it is not very difficult for us to understand that the function of p300 and its underling mechanism(s) to impact cancer progression may lead to this discrepancy. In addition, although we observed a positive association of p300 expression and Ki-67 expression (a marker for cell proliferation) in our HCC cohort, the precise signaling pathway that is ultimately involved in these processes remains to be investigated. However, our findings suggest a potential important role of *p300 *in the control of HCC cell proliferation, an activity that might be responsible, at least in part, for HCC tumorigenesis and/or progression.

Since advanced pTNM stage and tumor differentiation are the best-established risk factors for important aspects affecting the prognosis of patients with HCC [29]. These 2 parameters, based on specific clinicopathologic features and extent of disease, may have reached their limits in providing critical information influencing patient prognosis and treatment strategies. Furthermore, outcome of patients with same stage following surgery is substantially different and such large discrepancy has not been explored [30,31]. Thus, there is a need for new objective strategies that can effectively distinguish between patients with favorable and unfavorable prognosis. In this study, our results support the ideas that p300 expression, as examined by IHC, can identify patients with HCC that may show aggressive clinical course and poor outcome. Therefore, evaluation of p300 expression may become a biomarker for predicting prognosis and rendering a more tailored treatment strategy in patients with HCC. Based on the results, we propose a new prognostic model with high p300 expression, AFP levels and vascular invasion. This model including p300 expression can reflect the aggressive phenotype of HCC. Furthermore, its prognostic significance can be augmented by the elevated AFP levels and the presence of vascular invasion. Thus, this combined model may be a useful prognostic index for HCC.

## Conclusions

Our findings provide a basis for the concept that high expression of p300 may play an important role in the acquisition of an aggressive phenotype in HCC, suggesting that the expression of p300, as examined by IHC, will be a promising independent biomarker for shortened survival time of HCC patients. The combined clinicopathologic prognostic model may become a useful tool for identifying patients with different clinical outcomes.

## Abbreviations

AFP: alpha-fetoprotein; AUC: area under the curve; CBP: CREB binding protein; CREB: cAMP response element binding protein; HCC: hepatocellular carcinoma; Hulc: highly up-regulated in liver cancer; IHC: immunohistochemistry; ROC: receiver operating characteristic; TMA: tissue microarray.

## Competing interests

The authors declare that they have no competing interests.

## Authors' contributions

MYC is responsible for the study design. ML and RZL performed the experiments and draft the manuscript. JWC, JBL YC, JHH and QLW participated in the data analysis and interpretation. All authors read and approved the final manuscipt.

## References

[B1] Lau WY Lai EC Lau SH The current role of neoadjuvant/adjuvant/chemoprevention therapy in partial hepatectomy for hepatocellular carcinoma: a systematic review Hepatobiliary Pancreat Dis Int 2009 8 124 133 19357024

[B2] Jemal A Siegel R Ward E Hao Y Xu J Thun MJ Cancer statistics, 2009 CA Cancer J Clin 2009 59 225 249 10.3322/caac.20006 19474385

[B3] Cabibbo G Craxi A Epidemiology, risk factors and surveillance of hepatocellular carcinoma Eur Rev Med Pharmacol Sci 14 352 355 20496547

[B4] Frau M Biasi F Feo F Pascale RM Prognostic markers and putative therapeutic targets for hepatocellular carcinoma Mol Aspects Med 31 179 193 10.1016/j.mam.2010.02.007 20176048

[B5] Kundu TK Palhan VB Wang Z An W Cole PA Roeder RG Activator-dependent transcription from chromatin in vitro involving targeted histone acetylation by p300 Mol Cell 2000 6 551 561 10.1016/S1097-2765(00)00054-X 11030335

[B6] Vo N Goodman RH CREB-binding protein and p300 in transcriptional regulation J Biol Chem 2001 276 13505 13508 11279224 10.1074/jbc.R000025200

[B7] Goodman RH Smolik S CBP/p300 in cell growth, transformation, and development Genes Dev 2000 14 1553 1577 10887150

[B8] Muraoka M Konishi M Kikuchi-Yanoshita R Tanaka K Shitara N Chong JM Iwama T Miyaki M p300 gene alterations in colorectal and gastric carcinomas Oncogene 1996 12 1565 1569 8622873

[B9] Gayther SA Batley SJ Linger L Bannister A Thorpe K Chin SF Daigo Y Russell P Wilson A Sowter HM Mutations truncating the EP300 acetylase in human cancers Nat Genet 2000 24 300 303 10.1038/73536 10700188

[B10] Fan S Ma YX Wang C Yuan RQ Meng Q Wang JA Erdos M Goldberg ID Webb P Kushner PJ p300 Modulates the BRCA1 inhibition of estrogen receptor activity Cancer Res 2002 62 141 151 11782371

[B11] Bandyopadhyay D Okan NA Bales E Nascimento L Cole PA Medrano EE Down-regulation of p300/CBP histone acetyltransferase activates a senescence checkpoint in human melanocytes Cancer Res 2002 62 6231 6239 12414652

[B12] Fang Z Fu Y Liang Y Li Z Zhang W Jin J Yang Y Zha X Increased expression of integrin beta1 subunit enhances p21WAF1/Cip1 transcription through the Sp1 sites and p300-mediated histone acetylation in human hepatocellular carcinoma cells J Cell Biochem 2007 101 654 664 10.1002/jcb.21223 17211849

[B13] Wang J Liu X Wu H Ni P Gu Z Qiao Y Chen N Sun F Fan Q CREB up-regulates long non-coding RNA, HULC expression through interaction with microRNA-372 in liver cancer Nucleic Acids Res 38 5366 5383 10.1093/nar/gkq285 20423907 PMC2938198

[B14] Debes JD Sebo TJ Lohse CM Murphy LM Haugen DA Tindall DJ p300 in prostate cancer proliferation and progression Cancer Res 2003 63 7638 7640 14633682

[B15] Isharwal S Miller MC Marlow C Makarov DV Partin AW Veltri RW p300 (histone acetyltransferase) biomarker predicts prostate cancer biochemical recurrence and correlates with changes in epithelia nuclear size and shape Prostate 2008 68 1097 1104 10.1002/pros.20772 18459105 PMC3099408

[B16] Gao Q Qiu SJ Fan J Zhou J Wang XY Xiao YS Xu Y Li YW Tang ZY Intratumoral balance of regulatory and cytotoxic T cells is associated with prognosis of hepatocellular carcinoma after resection J Clin Oncol 2007 25 2586 2593 10.1200/JCO.2006.09.4565 17577038

[B17] Sobin LH Fleming ID TNM Classification of Malignant Tumors, fifth edition (1997). Union Internationale Contre le Cancer and the American Joint Committee on Cancer Cancer 1997 80 1803 1804 10.1002/(SICI)1097-0142(19971101)80:9<1803::AID-CNCR16>3.0.CO;2-9 9351551

[B18] Han L Lu J Pan L Wang X Shao Y Han S Huang B Histone acetyltransferase p300 regulates the transcription of human erythroid-specific 5-aminolevulinate synthase gene Biochem Biophys Res Commun 2006 348 799 806 10.1016/j.bbrc.2006.07.147 16904069

[B19] Cai MY Zhang B He WP Yang GF Rao HL Rao ZY Wu QL Guan XY Kung HF Zeng YX Xie D Decreased expression of PinX1 protein is correlated with tumor development and is a new independent poor prognostic factor in ovarian carcinoma Cancer Sci 101 1543 1549 10.1111/j.1349-7006.2010.01560.x 20367640 PMC11159430

[B20] Zlobec I Steele R Terracciano L Jass JR Lugli A Selecting immunohistochemical cut-off scores for novel biomarkers of progression and survival in colorectal cancer J Clin Pathol 2007 60 1112 1116 10.1136/jcp.2006.044537 17182662 PMC2014838

[B21] Ishihama K Yamakawa M Semba S Takeda H Kawata S Kimura S Kimura W Expression of HDAC1 and CBP/p300 in human colorectal carcinomas J Clin Pathol 2007 60 1205 1210 10.1136/jcp.2005.029165 17720775 PMC2095491

[B22] Karamouzis MV Konstantinopoulos PA Papavassiliou AG Roles of CREB-binding protein (CBP)/p300 in respiratory epithelium tumorigenesis Cell Res 2007 17 324 332 10.1038/cr.2007.10 17372613

[B23] Borrow J Stanton VP Jr Andresen JM Becher R Behm FG Chaganti RS Civin CI Disteche C Dube I Frischauf AM The translocation t(8;16)(p11;p13) of acute myeloid leukaemia fuses a putative acetyltransferase to the CREB-binding protein Nat Genet 1996 14 33 41 10.1038/ng0996-33 8782817

[B24] Kurebayashi J Otsuki T Kunisue H Tanaka K Yamamoto S Sonoo H Expression levels of estrogen receptor-alpha, estrogen receptor-beta, coactivators, and corepressors in breast cancer Clin Cancer Res 2000 6 512 518 10690532

[B25] Zhang C Guo X Jiang G Zhang L Yang Y Shen F Wu M Wei L CpG island methylator phenotype association with upregulated telomerase activity in hepatocellular carcinoma Int J Cancer 2008 123 998 1004 10.1002/ijc.23650 18546260

[B26] Fermento ME Gandini NA Lang CA Perez JE Maturi HV Curino AC Facchinetti MM Intracellular distribution of p300 and its differential recruitment to aggresomes in breast cancer Exp Mol Pathol 88 256 264 10.1016/j.yexmp.2010.01.007 20097195

[B27] De-Carvalho MC Chimelli LM Quirico-Santos T Modulation of fibronectin expression in the central nervous system of Lewis rats with experimental autoimmune encephalomyelitis Braz J Med Biol Res 1999 32 583 592 10.1590/S0100-879X1999000500012 10412570

[B28] Hudelist G Czerwenka K Kubista E Marton E Pischinger K Singer CF Expression of sex steroid receptors and their co-factors in normal and malignant breast tissue: AIB1 is a carcinoma-specific co-activator Breast Cancer Res Treat 2003 78 193 204 10.1023/A:1022930710850 12725419

[B29] Farinati F Rinaldi M Gianni S Naccarato R How should patients with hepatocellular carcinoma be staged? Validation of a new prognostic system Cancer 2000 89 2266 2273 10.1002/1097-0142(20001201)89:11<2266::AID-CNCR15>3.0.CO;2-0 11147597

[B30] Lau WY Lai EC Hepatocellular carcinoma: current management and recent advances Hepatobiliary Pancreat Dis Int 2008 7 237 257 18522878

[B31] Bruix J Sherman M Management of hepatocellular carcinoma Hepatology 2005 42 1208 1236 10.1002/hep.20933 16250051

